# JointCalc: A web-based personalised patient decision support tool for joint replacement

**DOI:** 10.1016/j.ijmedinf.2020.104217

**Published:** 2020-10

**Authors:** Evgeny Zotov, Andrew F. Hills, Fabio L. de Mello, Parham Aram, Adrian Sayers, Ashley W. Blom, Eugene V. McCloskey, J. Mark Wilkinson, Visakan Kadirkamanathan

**Affiliations:** a Department of Automatic Control and Systems Engineering, University of Sheffield, Sheffield, UK; b Department of Oncology and Metabolism, University of Sheffield, UK; c School of Clinical Sciences, University of Bristol, Bristol, UK

**Keywords:** Health information system (HIS), Decision support system, Medical informatics application, User-centered design, End-user participation, Agile software development

## Abstract

**Background and purpose:**

Health information systems (HIS) are expected to be effective and efficient in improving healthcare services, but empirical observation of HIS reveals that most perform poorly in terms of these metrics. Theoretical factors of HIS performance are widely studied, and solutions to mitigate poor performance have been proposed. In this paper we implement effective methods to eliminate some common drawbacks of HIS design and demonstrate the synergy between the methods. JointCalc, the first comprehensive patient-facing web-based decision support tool for joint replacement, is used as a case study for this purpose.

**Methods and results:**

User-centred design and thorough end-user involvement are employed throughout the design and development of JointCalc. This is supported by modern software production paradigms, including continuous integration/continuous development, agile and service-oriented architecture. The adopted methods result in a user-approved application delivered well within the scope of project.

**Conclusion:**

This work supports the claims of high potential efficiency of HIS. The methods identified are shown to be applicable in the production of an effective HIS whilst aiding development efficiency.

## Highlights

• JointCalc is the first complete web decision support tool for joint replacement.
• User-centred design helps avoid common health information system design.
• Modern software production methods synergise with and enable user-centred design.
• JointCalc implementation supports claims of high efficiency of eHealth.

## 1 Introduction

Hip and knee replacement are two of the most common elective operations, with over 100,000 of each performed annually in the UK [1]. Not all patients benefit from surgery. Between 10% and 20% suffer from moderate to severe long-term pain and a significant minority having severe complications that require repeat (revision) surgery or result in death [1]. Outcomes such as mortality and risk of revision surgery vary greatly according to patient factors, including age, gender, body mass index and co-morbidities [2], [3]. Modifiable treatment options such as surgical technique [4], use of thromboprophylaxis [5] and implant choice are also associated with outcomes [1]. It is thus critical that patients are equipped and empowered to make informed personalised decisions about their care.

**Fig. 1:**
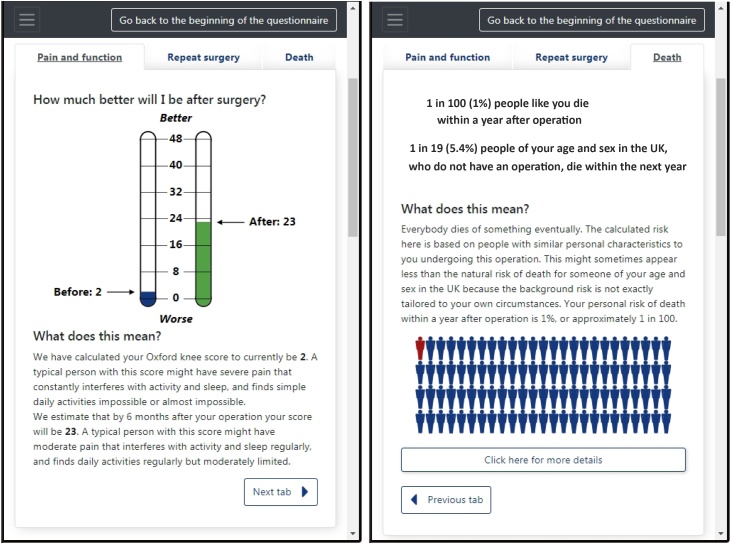
Sample results reported to a user by JointCalc.

JointCalc^2^ is the first comprehensive web-based patient decision support tool for joint replacement. The ultimate goal of JointCalc is to provide the patient, as the end-user, with the relevant information necessary to make weighted decisions, based on personalised risks and estimates of expected outcomes, in addition to general information about the operation (Fig. 1).

In JointCalc, the outcome of surgery is measured using three metrics: (1) expected patient reported outcome measure (PROM) score for joint pain and quality of life at six months post-surgery; (2) risk of death within one year post-surgery; (3) risk of the patient requiring revision surgery within ten years following the initial operation. A patient's post-operative PROM score is an integer quantity and is estimated as a function of the patient's attributes using a regression model. The risks of death and revision are the conditional probabilities of an event as a function of time and the patient's attributes, computed using survival analysis models.

The software delivered within the project is required to be scalable, easily accessible to a heterogeneous audience and future-proof beyond the initial development stage. A web-based solution has the potential to satisfy these needs with minimal overhead costs.

In spite of the high potential benefit of HIS implementation, poor usability of a deployed application is a common reason for its poor performance [6]. Although research shows that use case analysis is a crucial instrument for HIS application development [7], the industrial HIS implementation cases commonly direct resources to train users to understand an overcomplicated user interface, rather than to design an interface that facilitates end-usability [8], [9]. This distinction is of critical relevance if the end-user has limited software literacy. The level of involvement of the end-users during all stages of an HIS implementation project is thus a major factor influencing the return on the investment in the project [9], [10], [11], [12].

This work aims to describe the novel implementation of a personalised risk estimation methodology integrated in a HIS that is focussed on its end user, the patient. The algorithm framework that drives the web-enabled HIS interface incorporates personalised information and treatment choices. We achieved this aim by: (1) developing a web-enabled HIS that is centred on utility for the end user; (2) showing the integration of HIS development techniques that minimises common drawbacks of the established state-of-art methods, using JointCalc as a practical example; and (3) evaluating the performance of the tool in respect of its utility and usability, as assessed by end-user feedback.

## 2 Problem description

### 2.1 Design of health information systems

Health information systems (HIS) are proven to enhance the efficiency of a healthcare service by providing clinicians with timely and detailed information about the patient and serving the patient with a better understanding of the disease [13]. Despite estimating the high value of HIS deployment, empirical data poorly supports the theoretical usefulness and efficiency of these systems in healthcare [14]. 98% of the software developed for the US Government is considered to be “unusable as delivered”, only 61% of implemented HIS fulfil the requirements, and 63% consume costs above plan [15]. A review of HIS failings reveals that most of the errors stem not from technological issues, but from the adoption of design or development patterns that disregard the needs of the end-users [9]. Chiasson and Davidson [16] present evidence implying that the likely cause of HIS project failures lies in the early design phase, and the most common issue that results in poor system requirements satisfaction and project budget overruns is the low quality analysis of end-user requirements [15].

Approximately 80% of HIS maintenance costs are attributable to usability and user interface improvements [17]. Furthermore, remediation of errors uncovered during the application design phase is estimated to be 10 times less costly than of errors encountered during development [15], [17]. Therefore, maximisation of success probability for a HIS application implies that increased attention and resource should be devoted to the elicitation of user requirements and to the system design process at the initial stage of development. Agile design and development with frequent inclusion of the end-users into the software production workflow is consequently a key element of a successful design strategy for HIS application [18].

Black et al. [19], Ammenwerth et al. [14] assert that developers with little understanding of end-user needs will unlikely be able to incorporate them into the system. For example, where systems are initiated by clinical organisations that lack feedback from the patient side during their development, the result is poor performance outside of the institution they originated from [20]. An effective approach to bridge the gap between the developers and the end-users is user-centred design, an application design approach that aims to extensively involve the user from the earliest stages of the development process [19]. This approach includes analysis of user goals, requirements and behaviours into the design process of human–computer interactions [7]. Commencement of these tasks should precede the beginning of the application design phase [21]. Hence, potential end-users are integrally involved before and throughout the process of application design, whilst the software tool is evaluated against their needs and modified based on the user's feedback [18].

The underlying health need that JointCalc aims to address is the provision of personalised estimated outcome information for patients considering joint replacement. Patient consultation exercises conducted during the design phase of the tool showed that the key questions of interest to patients were:

1. How much better will my pain and function be after the operation?
2. What is my risk of dying after the operation?
3. What is my risk of needing revision surgery?
4. How does each risk vary with my personal biometric characteristics and the surgical choices that I make as a patient?

In order to develop algorithms that reliably underpin these questions across a broad patient demographic and a similarly large range of intervention choices, a large and detailed dataset of individual patient procedure episodes and their outcomes is required. This must then be integrated into a patient-facing tool that presents the choices in an accessible manner in order to achieve the required end-user utility.

### 2.2 Patient reported outcome measure

A PROM is a quantitative metric of a patient's perception of the pain and function of their affected joint. JointCalc provides the user with a comparison of their pre- and post-surgery PROM scores. The post-operative score is estimated using a linear regression model where the input variables are the patient's current biometrics and the pre-operative PROM score.

The patients are divided into two groups based on the joint surgery type: hip or knee. For each group, two models are constructed with different variable selections: a community-based model that uses basic biometric data of the patient, and a clinic-based model that includes potential surgical choices but necessitates assistance from a healthcare professional for data input.

### 2.3 Survival analysis

Two different events are studied in the experimental scenario for the JointCalc model: death and revision surgery following the initial surgical episode. Since the two events are not considered to be causally correlated, each are expressed separately, considering the other event as censored, which makes this choice equivalent to the assumption made in [22] for the case of a linear model.

Different mortality and revision models are used based on the type of operation and variable selection. Surgical operations reported in data from the National Joint Registry^3^ are consequently divided into four groups:

• hip replacement;
• total knee replacement;
• patellofemoral knee replacement;
• unicompartmental knee replacement.

For each group, both a community-based and a clinic-based model are built. The models developed include: revision risk modelling after total, patellofemoral or unicompartmental knee replacement [23]; revision risk modelling after hip replacement [24]; and mortality risk modelling for each of these patients groups [25].

## 3 Computational modelling

The PROM modelling used patient questionnaire data from NHS-Digital, including 319,030 hip and 338,672 knee replacement surgeries performed between May 2009 and February 2018. Modelling was performed using minimum mean square error estimation. The square of the age was included as part of the inputs in this model, resulting in the following formulation:

(1) $${\mathrm{PROM}}_{\mathrm{post}-\mathrm{op}}=\beta_{0}{\mathrm{PROM}}_{\mathrm{pre}-\mathrm{op}}+\beta_{1}\mathrm{Age}+\beta_{2}{\mathrm{Age}}^{2}+\sum_{k=3}^{K}\beta_{k}x_{k}$$

where $x_{k}$ are input variables other than PROM and age.

The estimations for the risks of death and revision were built using data from the National Joint Registry for England and Wales (NJR). After pre-processing and removal of procedures with incomplete records, the data includes 327,238 hip and 430,455 knee replacement surgeries performed between April 2003 and September 2015. The hip replacement data was used in a single model, whilst the knee replacement data were divided into 387,459 total; 37,693 unicompartmental; and 5,303 patellofemoral knee replacements, with a different revision model for each knee replacement type. The survival modelling was performed with the proportional hazards version of the flexible parametric model [26] that defines the cumulative hazard function as

(2) $$\Lambda (\text{x},t)=\Lambda_{0}(t)\exp(\beta \text{x}).$$

The proportionality factor exp(βx) is given by a log-linear model with x as inputs and β as parameters. The baseline cumulative hazard function is represented with natural cubic splines

(3) $$\Lambda_{0}(t)=\gamma_{0}+\gamma_{1}\log(t)+\sum_{j=1}^{m}\gamma_{j+1}\nu_{j}(\log(t)),$$

where $\gamma_{j}$ are parameters and $\nu_{j}(y)$ are the basis functions for the natural cubic splines, which are defined so that the resulting function is a third order polynomial in the middle interval and linear in the extreme intervals. The second derivative is constrained to be continuous between intervals. Parameters β and $\gamma_{j}$ were estimated jointly through maximum likelihood estimation as proposed in [26]. Algorithm 1 describes the implementation of this model in JointCalc. The data was filtered so that data from patients with input data incomplete or outside common ranges were not taken into account. The ASA rating was restricted to be either 1, 2, or 3; the age was restricted to interval from 30 to 100 years; and the BMI was restricted to the interval from 15 to 55.

Each model was validated with 50 repetitions of a fivefold cross validation procedure. In each repetition, the data was randomly partitioned into 5 subsets and estimation was performed with each combination of 4 subsets, with the remaining subset being used to compute the performance metrics. The performance metrics at each repetition was given by the average over the 5 different combinations, and the results for each repetition were used to compute their overall average with the 95% confidence interval. The results of the validation are presented in [23], [24], [25].

Algorithm 1 JointCalc PROM and risks calculation algorithm

**Table:** 

1: *request_data* ⟵ get(survey data)
2: *surg_type*, *model_type*, *dsver* ⟵ extract(joint type, model type, dataset version from *request_data*)
3: *prom_model*, *mort_model*, *reop_model* ⟵ *Model*(*surg_type*, *model_type*, *dsver*) ▷ Select the relevant models and load parameter coefficients
4: **x** ⟵ extract(patient biometrics, surgery options from *request_data*)
5: prom_score⟵prom_model(x)
6: exp (*β***x**)_mortality_ ⟵ *mort*_*model*(**x**)
7: **for***t* in *range*(number of years to estimate mortality risk) **do**
8: $\Lambda_{0}{\left(t\right)}_{\mathrm{mortality}}⟵$ baseline mortality hazard at time t
9: $\Lambda {\left(\text{x},t\right)}_{\mathrm{mortality}}⟵\Lambda_{0}{\left(t\right)}_{\mathrm{mortality}}\exp{\left(\beta \text{x}\right)}_{\mathrm{mortality}}$
10: exp (*β***x**)_reoperation_ ⟵ *reop*_*model*(**x**)
11: **for**t in *range* (number of years to estimate reoperation risk) **do**
12: $\Lambda_{0}{\left(t\right)}_{\mathrm{reoperation}}⟵$ baseline reoperation hazard at time t
13: $\Lambda {\left(\text{x},t\right)}_{\mathrm{reoperation}}⟵\Lambda_{0}{\left(t\right)}_{\mathrm{reoperation}}\;\;\;\exp{\left(\beta \text{x}\right)}_{\mathrm{reoperation}}$
14: return $prom\_score,\Lambda {\left(x,t\right)}_{mortality},\Lambda {\left(x,t\right)}_{reoperation}$

## 4 User-centred design of JointCalc

The design of the JointCalc application follows the user-centred design paradigm. It is based on detailed interactions with patients and their supporting clinicians, and further analysis and conversion of the received user feedback into user-friendly design. This joint work heavily influenced the design, content and architecture of the decision support system.

The end-users of JointCalc were involved in early A/B testing of the overall web-tool layout and interface design via a series of patient panel meetings. Participants included both patients that were considering, or had previously undergone, joint replacement surgery and clinicians working in the field of arthritis care. Participants provided feedback both within the group meetings and remotely, following each meeting. The patient panel were initially presented with a number of patient- and clinician-centric decision support and calculator websites for other medical problems, including diabetes and fracture risk. The websites were assessed on their aesthetics, ease of understanding the language used, data entry methods, and how results were conveyed. Feedback was scored into three broad categories: “disliked”, “indifferent”, and “liked”. Four mock websites were then constructed based on the feedback, focussing on maximising the features that the patient panel liked and minimising those features that they disliked. The websites lacked core functionality and, instead, only served to demonstrate the appearance, example text and questions, and type of data entry methods available.

Based on the patient panel feedback, two website designs were discarded. The remaining two were further developed, introducing functionality, incorporating feedback, and including a preliminary version of the results page. Additional iterations of development and patient panel feedback sessions were conducted, with comments broadened to include criteria such as site responsiveness.

### 4.1 Data input design

Many users feel burdened when tasked with filling a form, with services observing high user drop-out rates on such activities [27], [28]. A major part of the JointCalc design effort was thus focussed on increasing the comfort and speed of data input for the users. This included features to streamline the process and minimise the input actions required. For example, when a user completes a section of the form, the application automatically advances to the next section by unfolding the respective card element and scrolling the viewport to the target element. This relieves the user from any navigational interactions to facilitate data entry. Moreover, the flow between the form input fields allows the user to complete it from start to finish using only a single means of input, be it keyboard, mouse or a device's touch controls. Additionally, if a user returns to the questionnaire web page after successfully completing it once, they are presented with the opportunity to automatically restore the previously inputted data. An alternative way of interacting with the form is made available to service providers: the form can be pre-filled with data from the link that a user follows to arrive at the JointCalc web page.

### 4.2 Accessibility design

**Fig. 2:**
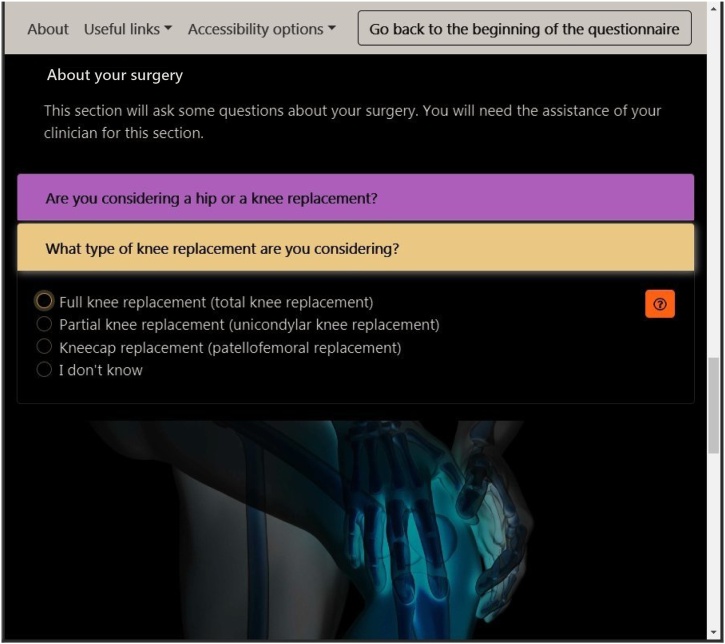
JointCalc with large font and high contrast options enabled.

Accessibility facilitates adoption, particularly in an older adult target demographic, and was therefore a focal point during JointCalc design. Patient panel feedback was invaluable for improving readability and usability of the interface materials. The various viewpoints were integrated to formulate the text in an optimally comprehensible manner for readers across different backgrounds and educational level. JointCalc also hosts functionality aimed at users with disabilities, including options that control font size and contrast of the colour scheme (see Fig. 2).

### 4.3 Personalisation

**Fig. 3:**
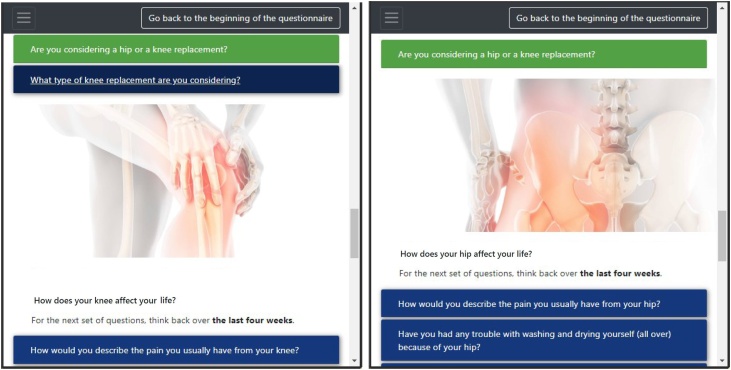
Adaptive JointCalc content: for knee replacement patients (left) and hip replacement (right).

Sillence et al. [29] propose that user acceptance of healthcare web services is hampered by users’ inability to identify with the provided content, which is sometimes expressed as a corporate or bureaucratic feel to the website. Mitigations to this problem include social identification and personalisation of the content. To achieve this, JointCalc tailors the experience for a specific user and presents personalised information by adapting the content and text based on the user's preferences. Fig. 3 depicts an example from the form page, illustrating the variability in text and imagery for users considering surgery for different joint types.

## 5 JointCalc development approach

**Fig. 4:**
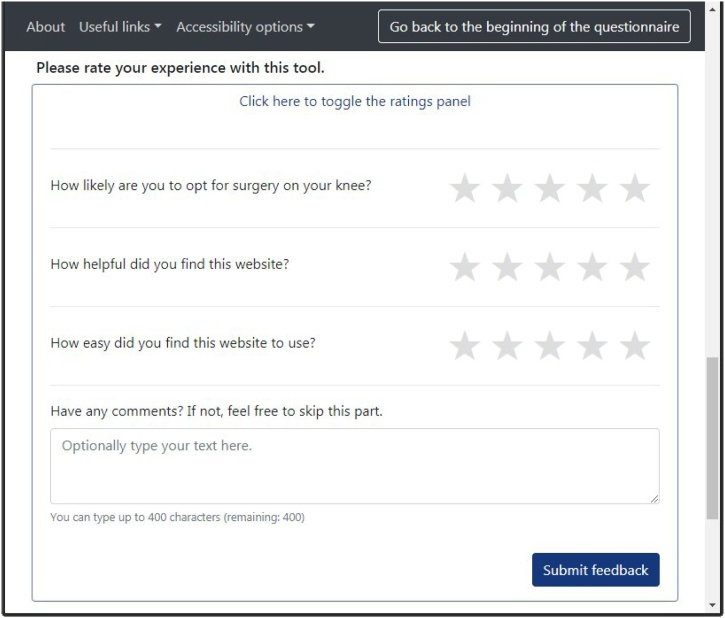
JointCalc feedback form.

JointCalc development had to accommodate both the chosen iterative design and the long-term implementation goals. These implications conform to many modern software implementation requirements and are largely tackled through an agile [30] development approach and an adoption of a continuous integration/continuous deployment (CI/CD) tool set. Agile shifts the focus from planning, processes and standardisation to flexibility, people and customisation. Thorough user inclusion in the development process is also highly encouraged [31]. In addition to the patient panel meetings prior to the live release of JointCalc, ongoing tool evolution is supported by a feedback facility built into the website that is aimed at capturing the key outcomes of JointCalc use: user's next steps, subjective JointCalc usability and content quality (see Fig. 4).

Automated testing is the main tool that enables agile development for JointCalc. Automated tests cover the key functional (i.e. interactive elements functioning, back-end logic and interactions, etc.) and usability (i.e. text visibility, responsive layout, etc.) features of the tool and are executed after every code modification. This achieves an increase in the robustness of service quality, whilst simultaneously focussing the development effort on enhancement rather than maintenance. Container management automation also further reduces project costs by streamlining the creation, updating and deployment of test and development servers. An approximation based on the number of commits and live releases yields an efficiency gain of 334 h (more than eight full-time weeks at 40 h effort per week) during one year of development due to test automation.

The flexible development approach could be partly inhibited by a monolithic application architecture, as small adjustments to the code base produce significant overheads, as for example, in large amounts of regression testing. Moreover, the upgrades of a single large application typically take more time than atomic updates of smaller components, resulting in higher application downtime. A service-oriented architecture (SOA) is an application architecture approach that addresses the issues stated above, and promotes segregation of application functionality into loosely coupled parts where each functional module is considered a service. Such a modular architecture aims to divide the code base into more manageable pieces and reduce the interdependence between the application's functional parts, thus enabling desynchronised and incremental upgrades of the separated services [32]. These advantages of SOA are crucial for agile development, as they reduce the costs introduced by the flexibility requirements. A microservices architecture is a modern refinement of SOA that advises a further size reduction of the developed services and takes advantage of the containerised deployment toolkit. Microservices extend the SOA paradigm in promoting the increased evolvability, decentralisation and automation of the processes within the developed software components [33,34].

The JointCalc application architecture design is guided by the application requirements and development processes with future scalability and maintainability being the prime drivers. To meet these requirements, JointCalc adopts the microservices architecture design approach. The application is split into services based on the maintained functional areas.

**Fig. 5:**
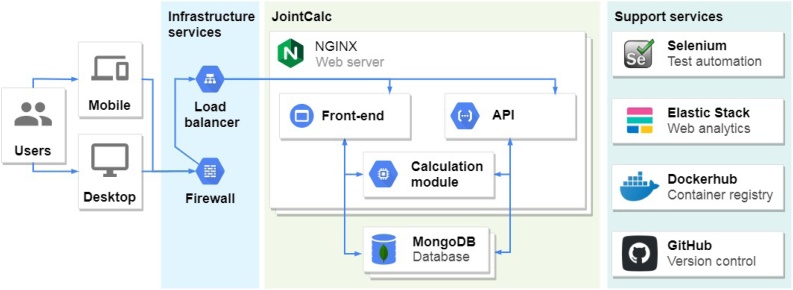
JointCalc application architecture.

The following list presents a brief enumeration of the components of JointCalc architecture depicted in Fig. 5:

• Load-balancer receives external requests over HTTPS and distributes them among available service instances that execute the relevant functionality.
• Web server handles the incoming HTTP requests and serves either HTML pages or Application programming interface (API) response messages depending on the content of the received HTTP request.
• Front-end service produces the HTML pages served to a user's device.
• API service exposes the back-end services for programmatic access.
• The calculation module encapsulates the implementation logic of the models that produce the risk and patient outcome estimates.
• The database service accompanies the web server and provides data storage and retrieval functionality.
• Web analytics service generates insights from the web server access logs.
• Test automation service executes the automated test suites and produces testing reports.
• Container registry is used to share the containerised environment configurations.
• Version control system provides version tracking and general project planning features.

In addition to the previously highlighted efficiency benefits for agile development, the modularity of microservices significantly enhances the maintainability of the code base. Meaningful distribution of code among the services provides the developers with a clear map of application functionalities with reduced amount of documentation, thus easing both new developer onboarding and routine debugging. The choice of specific technologies used for JointCalc is primarily guided by domain-specific requirements, taking into account component performance estimates and project cost optimisation.

## 6 JointCalc public use and feedback evaluation

The success of the implementation is reflected in JointCalc usage and the feedback from its users recorded to date of writing (the first nine months after public deployment). From a total of 15,372 unique visitors to the website, 5120 have viewed the results. 8375 of the unique visitors were from the UK and the rest were from 110 other countries around the world.

33,103 non-unique visits to the results page resulted in 2994 feedback submissions via the feedback form (Fig. 4). From this feedback, the mean usefulness score (scored out of a total of 5) was 4.4 (standard deviation 1.0) and mean ease of use was 4.7 (0.8). The patients’ responses frequently included phrases indicating that the website helped them reach a decision about the surgery. Free text comments included feedback that the tool is “informative”, “helpful” and “easy to use”. Both the quantitative metrics and the qualitative responses indicate that the users find JointCalc user-friendly and relevant to their problem.

## 7 Conclusion

JointCalc is a practical example of an effective software implementation of a HIS, and represents the first comprehensive web-based patient decision support tool for joint replacement. The developers utilise existing knowledge of pitfalls common in HIS implementations and leverage the solutions proposed by the scientific community to avoid them. This approach impacts the design and development paradigms followed throughout the project, resulting in an application design that supports presentation of information in a comprehensive manner that demands neither prior understanding of the problem nor training in application use. The tool thus exposes the complexity of a novel computational framework in a user-friendly way available to a wide audience. The chosen approach also shapes the production process, demanding high efficiency that takes form in an automated testing system. This not only makes the project more cost-effective, but also enables the fast and efficient processing of acquired feedback.

## Summary table

What was already known on the topic?

• Health information systems are expected to be effective and efficient in improving healthcare services.
• However, most perform poorly either in terms of efficiency or effectiveness, or both. The main causes of this inefficiency have been established.
• User-centred design is an application design approach that may counter the known drawbacks of traditional application design methods.

What did this study add to our knowledge?

• We describe the development of the first web-enabled, personalised, patient-centred decision support tool of user-centred design.
• We show that user-centred design can be practically applied to produce an effective health information system, while also improving its development efficiency.
• Modern software design paradigms, such as agile and service-oriented architecture, organically fit the processes implied by adoption of user-centred design approach.

## Author contributions

JMW and EVM initiated the JointCalc concept, JMW led the development process. EZ and AFH developed the application throughout the case study, supervised by VK and JMW. AFH architected the application and administrates the system. EZ developed the application monitoring and CI/CD stacks. PA and AS worked on the risk modeling methodology, supported by JMW and AB. EZ wrote the initial version of the paper and edited it after review, AFH wrote the initial application design, FLM added the background on risk modelling methodology. All authors reviewed and edited the paper prior to submission.

## Conflict of interest

None declared.

## Footnotes

2 Patient Decision Support Tool for Joint Replacement, available at https://jointcalc.shef.ac.uk/.

3 More information on National Joint Registry at http://www.njrcentre.org.uk.
